# Aberrant Brain Network Integration and Segregation in Diabetic Peripheral Neuropathy Revealed by Structural Connectomics

**DOI:** 10.3389/fnins.2020.585588

**Published:** 2020-12-04

**Authors:** Fangxue Yang, Minli Qu, Youming Zhang, Linmei Zhao, Wu Xing, Gaofeng Zhou, Jingyi Tang, Jing Wu, Yuanchao Zhang, Weihua Liao

**Affiliations:** ^1^Department of Radiology, Xiangya Hospital, Central South University, Changsha, China; ^2^Department of Endocrinology, Xiangya Hospital, Central South University, Changsha, China; ^3^Key Laboratory for NeuroInformation of Ministry of Education, School of Life Sciences and Technology, University of Electronic Science and Technology of China, Chengdu, China; ^4^Molecular Imaging Research Center of Central South University, Changsha, China; ^5^National Clinical Research Center for Geriatric Disorders (XiangYa), Changsha, China

**Keywords:** cortical thickness, diabetic peripheral neuropathy, graph theory, structural covariance networks, integration, segregation

## Abstract

Diabetic peripheral neuropathy (DPN) is one of the most common forms of peripheral neuropathy, and its incidence has been increasing. Mounting evidence has shown that patients with DPN have been associated with widespread alterations in the structure, function and connectivity of the brain, suggesting possible alterations in large-scale brain networks. Using structural covariance networks as well as advanced graph-theory-based computational approaches, we investigated the topological abnormalities of large-scale brain networks for a relatively large sample of patients with DPN (*N* = 67) compared to matched healthy controls (HCs; *N* = 88). Compared with HCs, the structural covariance networks of patients with DPN showed an increased characteristic path length, clustering coefficient, sigma, transitivity, and modularity, suggestive of inefficient global integration and increased local segregation. These findings may improve our understanding of the pathophysiological mechanisms underlying alterations in the central nervous system of patients with DPN from the perspective of large-scale structural brain networks.

## Introduction

Diabetic peripheral neuropathy (DPN), as one of the most common forms of peripheral neuropathy, affects approximately 30–50% of the diabetic population worldwide. Typical clinical manifestations of this disease include positive sensory symptoms in the feet, such as tingling, prickling, and pain, as well as negative symptoms, such as numbness, leading to considerable disability and suffering (Feldman et al., 2017). Although DPN has long been deemed as a disease solely of the peripheral nervous system, mounting evidence has suggested that central nervous system abnormalities also play important roles in the maintenance and development of this disease (Selvarajah et al., 2011, 2014b; West et al., 2015; Halb et al., 2017). Indeed, patients with DPN have been associated with widespread alterations in the structure (Selvarajah et al., 2014b; Zhang et al., 2020), function (Tseng et al., 2013; Li et al., 2018; Segerdahl et al., 2018; Venkataraman et al., 2019) and connectivity of the brain (Cauda et al., 2009a,b, 2010; Cui et al., 2015; Segerdahl et al., 2018), suggesting possible alterations in large-scale brain networks.

Graph theory offers a useful tool for characterizing the topological organization of large-scale brain networks. Using graph theoretical approaches, previous studies have shown that the brain networks of healthy subjects possess an economical small-world topology (i.e., high clustering coefficient and low path length), an architecture that enables both the specialization and the integration of distributed networks at low wiring costs (Bullmore and Sporns, 2009). In previous neuroimaging studies, altered small-world topology has been observed in various brain diseases, such as Alzheimer’s disease, schizophrenia, amyotrophic lateral sclerosis, and attention-deficit/hyperactivity disorder, reporting a suboptimal lattice-like network organization with an increased clustering coefficient and an increased path length (He et al., 2008; Zhang et al., 2012, 2019; Cao et al., 2013). Networks as such have been shown to be associated with reduced signal propagation speed and synchronizability compared with small-world networks, resulting in less efficiency in global integration (Strogatz, 2001). Of note, studies in some pain conditions demonstrated aberrant topological properties, such as an increased clustering coefficient and a decreased local efficiency (Liu et al., 2012, 2018), suggesting that long-term peripheral nociceptive input could have an effect on the topology of large-scale brain networks. Therefore, given that DPN is usually associated with neuropathic pain, one may expect topological alterations in the brain networks in patients with DPN, such as an increased clustering coefficient and decreased efficiency parameters.

In recent years, gray matter structural covariance, defined as the correlation of some morphological index between pairs of brain regions, has offered a useful means to construct large-scale structural brain networks (i.e., structural covariance networks). A key assumption of this method is that morphological correlations are related to axonal connectivity between brain regions with shared trophic, genetic, and neurodevelopmental influences (Alexander-Bloch et al., 2013). It has been demonstrated that structural covariance networks correspond well with functional networks and tractography-based white matter networks within the framework of graph theoretical analysis (Hosseini et al., 2016; Bruno et al., 2017). By contrast, the construction of structural covariance networks needs relatively lower computational loads and is arguably less sensitive to noise (Bethlehem et al., 2017). To the best of our knowledge, no study to date has examined the structural covariance networks in DPN. Given that previous studies have documented significant alterations in gray matter morphology in patients with DPN (Zhang et al., 2020), structural covariance network analysis that assesses the coordination patterns of gray matter morphology may provide new insights into the pathophysiology of this disease.

In the present study, we aimed to investigate the topological abnormalities of structural covariance networks for a relatively large sample of patients with DPN (*N* = 67) compared to matched healthy controls (HCs; *N* = 88). Specifically, by assessing the interregional correlation of cortical thickness, we constructed the structural covariance networks for each group. Several global network parameters, such as small-world indices, modularity and efficiency measurements, as well as a few regional network parameters, such as the nodal degree and nodal clustering coefficient, were then extracted and compared between the two groups. We hypothesized that the structural covariance networks of patients with DPN would show altered global network parameters such as an increased path length and clustering coefficient, overall suggestive of a less integrated yet more segregated network organization.

## Materials and Methods

### Subjects

Sixty-seven type-2 diabetes mellitus patients with a diagnosis of DPN and 88 HCs were recruited consecutively from Xiangya Hospital, Central South University (see Zhang et al., 2020, Hum Brain Mapp). The diagnosis of DPN was made according to the American or Toronto consensus criteria (Boulton et al., 2011). The inclusion criteria for DPN patients were as follows: right-handed; an age range from 30 to 68 years; and stable glycemic control (HbA1c 9.36 ± 2.14%). Patients were excluded from the study if they had (1) other diabetic neuropathies or nondiabetic neuropathies; (2) hypoglycemic unawareness; (3) neurological, psychiatric or cerebrovascular diseases; (4) prior substantial head trauma or tumors; or (5) alcoholism or drug abuse. The inclusion criteria for the HCs were as follows: (a) no chronic pain conditions or analgesic medications for treatment of pain; (b) no history of head trauma, surgery or brain tumors; (c) absence of neurological and psychiatric diseases; (d) no alcoholism or drug abuse. Demographic details and clinical assessment of all participants are shown in Table 1. This prospective study was approved by the Medical Research Ethics Committee of Xiangya Hospital, Central South University, and written informed consent was obtained from all subjects.

**TABLE 1:** Demographic data of the participants.

	**DPN patients (*N* = 67)**	**HC (*N* = 88)**	***P*-value**
Mean age in years (SE)	56.076(1.03)	55.580(0.83)	0.654
Male/female	39/28	56/32	0.472
HbA1c (%) [mmol/mol]	9.359 (2.135)	−	−
BMI	23.585 (3.241)	−	−
Duration of diabetes	8.202 (5.377)	−	−
NSS	4.240 (3.003)	−	−
NDS	1.800 (1.698)	−	−
DN4	2.290 (2.452)	−	−

*SE, standard error; BMI, body mass index; NSS, neuropathy symptom score; NDS, neuropathy disability score; DN4, douleur neuropathique en 4 questionnaire. Continuous variables were analyzed with independent two-sample t-test; categorical data were analyzed with a Chi-squared test. The significance level for the comparisons was set at p < 0.05.*

### MRI Analysis

#### MRI Data Acquisition

Three-dimensional T1-weighted MRI scans were obtained on a 3.0 T Siemens Magnetom Prisma MR system using a magnetization-prepared rapid acquisition gradient echo (MPRAGE) sequence. Detailed scan parameters were as follows: repetition time = 2,300 ms, echo time = 2.98 ms, inversion time = 900 ms, flip angle = 9°, thickness = 1.0 mm, no gap, 176 sagittal slices, field of view = 256 mm × 256 mm, matrix = 256 × 256, voxel size = 1.0 × 1.0 × 1.0 mm^3^, and sequence scan time = 5.2 min.

#### Cortical Thickness Measurement

The T1-weighted MRI scan of each subject was preprocessed using the FreeSurfer package (version 6.0.0) with its standard preprocessing pipelines (Wang et al., 2018). Briefly, an initial surface was derived through the segmentation of the white matter and the tessellation of the gray/white matter boundary. Through the performance of an automated topological correction operation, the initial surface was further refined to yield a topologically correct gray/white matter surface, which hereafter was referred to as the white surface. Subsequently, the white surface was deformed outward using a deformable surface algorithm to identify the pial surface. For all subjects, both the pial surface and the white surface were visually inspected for errors and manually corrected according to the software guidelines if necessary. After the generation of these surface models, the cortical thickness of each subject was obtained by calculating the distance between the white matter surface and the pial surface using the T-average algorithm. Before statistical analysis, the cortical thickness maps were resampled onto the standard space (fsaverage template).

### Network Analysis

#### Construction of Structural Covariance Networks

The cortical thickness map of each subject was parcellated into 68 brain regions (with 34 regions on each hemisphere) according to the Desikan-Killiany atlas^1^. The average cortical thickness of each cortical region was extracted and taken as the cortical thickness for the corresponding region. A linear regression analysis was performed for each cortical region to remove the effects of age, sex and global mean cortical thickness. The residuals of this regression, hereafter referred to as corrected cortical thickness, were used to construct structural covariance networks. For each group, a 68 × 68 correlation matrix, *R* = [*r*_*ij*_] (*i, j = 1, 2… N*, here *N* = 68), was obtained by computing Pearson’s correlation coefficients across individuals between the corrected cortical thickness of every pair of regions. Subsequently, the correlation matrix was thresholded to obtain a binary adjacency matrix, *A* = [*a*_*ij*_] (*i, j = 1, 2,… N*, here *N* = 68), where *a*_*ij*_ was retained as an edge (set equal to 1) if *r*_*ij*_ was greater than a threshold T, and *a*_*ij*_ was not retained (set equal to 0) if *r*_*ij*_ was less than T. Here, the threshold T was always greater than 0; thus, negative values for *r*_*ij*_ were set equal to 0. To remove self-loops, the diagonal elements of the binary adjacency matrix were set equal to 0. The resultant adjacency matrix *A* represented a binary undirected graph G (N,E). Here, N is the number of nodes which represent brain regions and E is the number of edges which represent undirected links between nodes corresponding to the nonzero elements in *A*.

#### Network Parameters

Our study extracted and compared some global and regional network parameters between the two groups, such as small-world properties, network efficiency, transitivity, modularity and nodal characteristics (Rubinov and Sporns, 2010).

##### Small-World Properties

The clustering coefficient, path length, gamma, lambda, and sigma are commonly used parameters for quantifying the small-world topology of a network. Briefly, the clustering coefficient of a node is defined as the ratio of the number of existing edges to the number of all possible edges that are directly adjacent to the node. The clustering coefficient of a network is defined as the average of clustering coefficients across nodes and reflects network segregation. The shortest path length between two nodes is defined as the least number of edges that separates them. The characteristic path length of a network is defined as the average shortest path length that connects any two nodes and reflects network integration. To examine the small-world properties, the clustering coefficient and characteristic path length are normalized to the corresponding mean values of matched random networks (Maslov and Sneppen, 2002). The small-world index (sigma) can then be obtained as the ratio of the normalized clustering coefficient (gamma) to the normalized characteristic path length (lambda).

##### Network Efficiency, Transitivity, and Modularity

The global efficiency is the inverse of the harmonic mean of the shortest path lengths across nodes in a network and is one of the most elementary indicators of integration. Local efficiency is defined as the average inverse shortest path length between a node and its direct neighbors and indicates the efficiency of information transfer within neighborhoods. The transitivity is similar to the clustering coeficient, but it is normalized by the whole network rather than by each node. Thus, the transitivity is not affected by nodes with a low degree. The modularity quantifies the degree to which a network is decomposed into subdivisions (modules) with maximal within-module connections and minimal between-module connections (Meunier et al., 2010).

##### Nodal Characteristics

The nodal degree, nodal clustering coefficient and nodal betweenness centrality were used to identify the regional alterations in the structural covariance networks. Nodal degree is defined as the number of connections that a node has with rest nodes of the network, which is considered a measure of the node’s interaction within the network. Nodal betweenness centrality is defined as the number of shortest paths between any two nodes in the network that pass through a given node. Before group comparison, the nodal degree, nodal clustering coefficient and nodal betweenness centrality were normalized by the average network degree, clustering coefficient and betweenness centrality, respectively. Hubs of a network are nodes that play a pivotal role in the control of information flow within the network. In our study, a node was considered a hub if its nodal betweenness centrality was at least 1.5 standard deviations higher than the average network betweenness centrality.

### Statistical Analysis

#### Cortical Thickness

Vertex-wise contrasts of cortical thickness maps were performed between DPN patients and HCs using the SurfStat package^2^. Specifically, each contrast was entered into a vertex-wise generalized linear model (GLM) with group, sex, age, and intracranial volume (ICV) as covariates. The results were first thresholded vertex-wise at *p* < 0.005 and then corrected for multiple comparisons at the cluster level using random field theory (RFT). The significance level for clusters was set at *p* < 0.05 after multiple-comparison correction. It should be noted that the individual cortical thickness map was smoothed with a 20 mm heat kernel prior to the statistical analysis.

#### Network Parameter Differences

All network parameters were compared between the two groups using the graph analysis toolbox (Hosseini et al., 2012). In this study, a wide range of network densities, namely, 0.15 ≤ D ≤ 0.40, was chosen for subsequent network analyses. The lower limit of the range was the minimum value where the networks of both groups were not fragmented. The upper limit of the range was the maximum value where the networks of both groups had a small-world index > 1.2 (Hosseini et al., 2013). Then, non-parametric permutation testing (1,000 repetitions) was used to test the statistical significance of the DPN-related differences in global and regional network parameters. Briefly, the network parameters were calculated for each network at each density. Between-group differences in the network parameters were then calculated to create a permutation distribution of difference under the null hypothesis. For each network parameter, the actual difference between DPN patients and HCs was placed in the corresponding permutation distribution to obtain the significance level. Furthermore, the area under the curve (AUC) was computed as a summary metric to evaluate the overall group-level differences across all densities. The significance level was set at *P* < 0.05 for group differences in global and regional network parameters.

## Results

### Decreased Cortical Thickness in DPN

**FIGURE 1:**
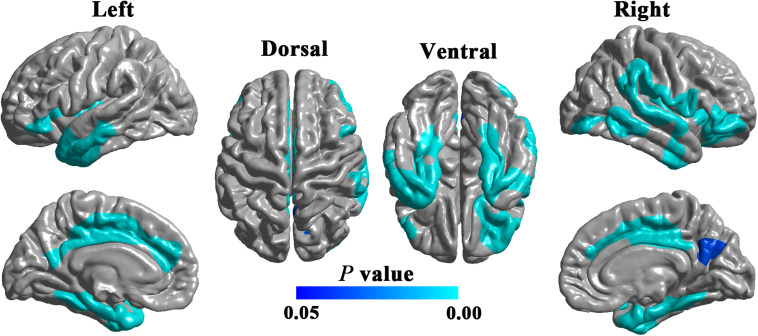
Cortical regions showing significantly decreased cortical thickness in DPN patients compared with HCs (RFT-corrected *P* < 0.05).

Compared with HCs, patients with DPN showed significantly decreased cortical thickness in widespread cortical regions (Figure 1). According to the Desikan-Killiany atlas, these regions involved the bilateral insular cortex; inferior frontal gyrus (including the pars orbitalis and pars triangularis) and lateral orbitofrontal gyrus; posterior and middle cingulate gyri; precuneus; superior frontal gyrus; inferior, middle, and superior temporal gyri; fusiform and parahippocampal gyri; entorhinal cortex; right post- and precentral gyri; and right supramarginal gyrus.

### Global Network Analysis

**FIGURE 2:**
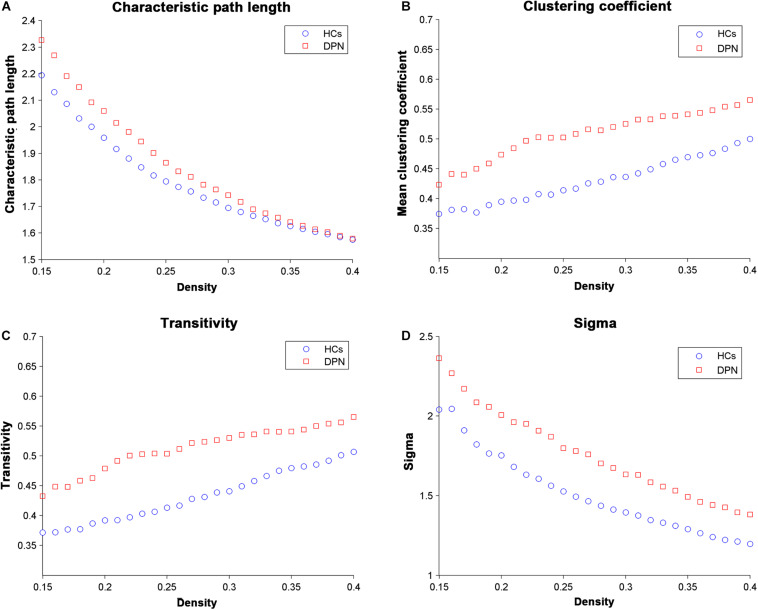
Changes in global network parameters as a function of network density. **(A)** Characteristic path length, **(B)** clustering coefficient, **(C)** transitivity, and **(D)** sigma in HCs and in DPN patients.

**FIGURE 3:**
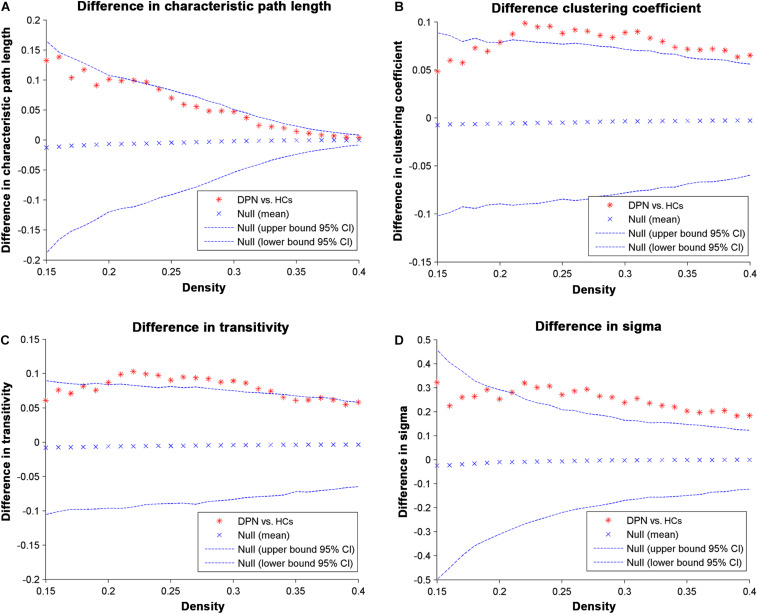
Differences between HCs and DPN patients in global network parameters as a function of network density. The 95% confidence intervals and group differences in the **(A)** characteristic path length, **(B)** clustering coefficient, **(C)** transitivity, and **(D)** sigma. The ^∗^ marker denotes the difference between HCs and DPN patients; the ^∗^ signs lying outside of the confidence intervals indicate the density where the difference is significant at *P* < 0.05. The positive values indicate DPN patients > HCs, and negative values indicate DPN patients < HCs.

Network parameters of the structural covariance networks for the two groups were calculated at a range of network densities (0.15–0.40) (Figure 2), which yielded fully connected networks with sigma > 1.2. For only a few network densities, the structural covariance networks of patients with DPN had a higher characteristic path length, clustering coefficient, transitivity, sigma (Figure 3), lambda, gamma (Supplementary Figure S1), and local efficiency and a lower global efficiency (Supplementary Figure S2) than those of HCs. Summary AUC analyses of these indices revealed a significantly increased characteristic path length (*P* = 0.0410), clustering coefficient (*P* = 0.0190), transitivity (*P* = 0.0220), lambda (*P* = 0.0390), gamma (*P* = 0.0060), sigma (*P* = 0.0060), and local efficiency (*P* = 0.026) and decreased global efficiency (*P* = 0.037) in the structural covariance networks of patients with DPN compared with HCs.

**FIGURE 4:**
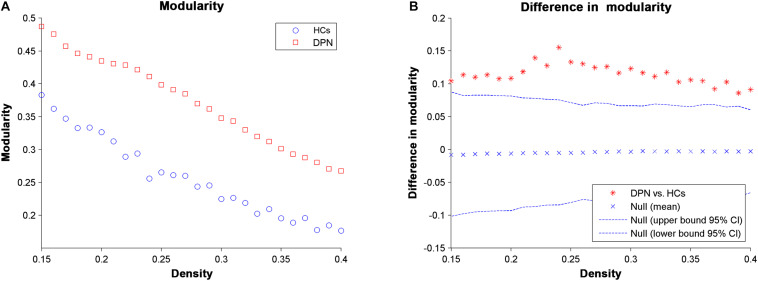
Changes in modularity **(A)** and between-group differences in modularity **(B)** as a function of network density. The ^∗^ marker denotes the difference between HCs and DPN patients; the ^∗^ signs lying outside of the confidence intervals indicate the density where the difference is significant at *P* < 0.05.

For all the network densities, the structural covariance networks of patients with DPN had higher modularity than those of HCs (Figure 4). Subsequent AUC analysis revealed a significant modularity increase (*P* = 0.003) in the structural covariance networks of patients with DPN compared with HCs.

### Regional Network Analysis

**FIGURE 5:**
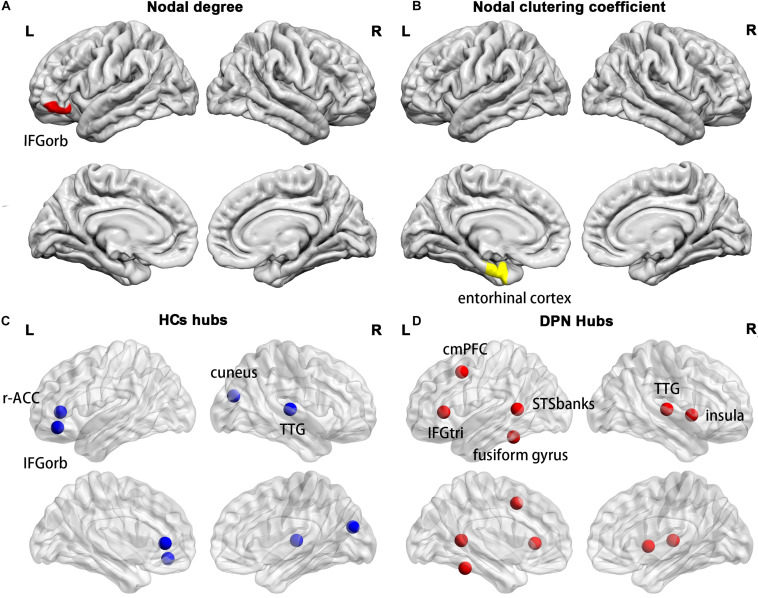
Cortical regions with a decreased nodal degree in the left pars orbitalis **(A)** and a decreased nodal clustering coefficient in the left entorhinal cortex **(B)** in DPN patients compared with HCs. The distribution of network hubs in HCs **(C)** and DPN patients **(D)**. IFGorb, inferior frontal gyrus pars orbitalis; r-ACC, rostral anterior cingulate cortex; TTG, transverse temporal gyrus; cmPFC, caudal middle prefrontal gyrus; IFGtri, inferior frontal gyrus pars triangularis; STSbanks, banks of the superior temporal sulcus.

Compared with HCs, we found a decreased nodal degree in the left pars orbitalis (Figure 5A) and a decreased clustering coefficient in the left entorhinal cortex (Figure 5B) in patients with DPN.

### Network Hub Analysis

Compared with HCs, a different number and distribution of network hubs were found in patients with DPN. Specifically, we identified more hubs in the DPN group (six hubs) than in the HC group (four hubs). The four hubs in the HC group included the left pars orbitalis, the left rostral anterior cingulate cortex, the right cuneus and the right transverse temporal gyrus (Figure 5C). The six hubs in the DPN group included the left banks of the superior temporal sulcus, the left caudal middle prefrontal gyrus, the left fusiform gyrus, the left pars triangularis, the right transverse temporal gyrus and the right insula cortex (Figure 5D).

## Discussion

By assessing the interregional correlation of cortical thickness, the present study investigated the topological abnormalities of the large-scale structural brain networks in DPN. Compared with HCs, the structural covariance networks of patients with DPN showed an increased characteristic path length, clustering coefficient, sigma, transitivity, and modularity, suggestive of inefficient global integration and increased local segregation. In addition to the alterations in global network parameters, regional network parameters of some brain areas were affected, showing a decreased nodal degree and nodal clustering coefficient in the patients with DPN compared with HCs. In addition, we also found that the number of hubs in the patients with DPN increased and anatomical location shifted, mainly in pain and cognition-related brain regions. These findings may improve our understanding of the pathophysiological mechanisms underlying alterations in the central nervous system of patients with DPN from the perspective of large-scale structural brain networks.

### Decreased Cortical Thickness in DPN Patients

Compared with HCs, patients with DPN showed significantly decreased cortical thickness mainly in some sensorimotor and pain-related brain regions. These findings are consistent with previous studies on DPN, showing cortical volume reduction in the postcentral gyrus and cingulate cortex and widespread cortical thickness reduction in the insula, cingulate cortex, prefrontal cortex and precentral gyrus (Selvarajah et al., 2014b; Zhang et al., 2020). Morphological alterations in these brain regions might be the anatomic substrates underlying the sensorimotor and pain-related impairments in DPN.

### DPN-Related Alterations in Global Network Parameters

The structural networks in both the DPN and HC groups displayed the small-world configuration, characterized by high clustering coefficients and low characteristic path lengths linking individual network nodes. However, compared with HCs, the structural networks of DPN patients showed increased characteristic path length, increased clustering, increased transitivity, increased local efficiency, and decreased global efficiency. The finding of increased characteristic path length and decreased global efficiency in patients with DPN may indicate impaired global integration, and is supported by our previous DTI study, showing widespread FA decreases throughout white matter tracts in patients with DPN (Zhang et al., 2020). In fact, increased characteristic path length and decreased global efficiency has been shown to be associated with decreased long-range connections (He et al., 2008, 2009). Since long-range connections are believed to form the basis of many cognitive processes (He et al., 2009; Meijer et al., 2020), and the previous clinical study have shown that the patients of DPN exhibited disturbances in visuospatial, verbal, and multi-tasking aspects of executive function, which is a group of higher cognitive control processes (Rucker et al., 2014). We therefore speculate that the observed increase in characteristic path length and decrease in global efficiency may underlie the abnormal cognitive functions in DPN (Cui et al., 2015). Meanwhile, increased local features is associated with the increased short-range connections among neighboring regions, suggested that the information processing is traversing more restrictedly within a clique of densely interconnected regions, i.e., an abnormally strong local segregation. Previous study showed that networks with increased local characteristics have better fault tolerance ability in the face of external attacks (Latora and Marchiori, 2001). In this work, the increased local segregation in the DPN group might reflect a compensatory action to suppress the influence of disease on the brain networks. Taken together, alterations in global parameters indicate that the topological organization of the brain networks in DPN patients is suboptimal, manifesting as inefficient global integration and increased local segregation.

### DPN-Related Alterations in Regional Network Parameters

Compared with HCs, we found a decreased nodal degree in the left pars orbitalis and a decreased clustering coefficient in the left entorhinal cortex in DPN patients. Since the present study showed decreased cortical thickness in the left pars orbitalis and entorhinal cortex, the decreased nodal degree and clustering coefficient may arise from the decreased cortical thickness of the two regions. Functionally, the pars orbitalis is part of the orbitofrontal network, which is associated with emotion processing in general and specifically with encoding the significance and value of stimuli (Du et al., 2020). The entorhinal cortex is an important structure of the medial temporal lobe and plays a key role in the interaction between the neocortex and the hippocampus in support of declarative and spatial memory (Piguet et al., 2018). Indeed, previous studies have reported that the prevalence of mood disorders in DPN patients is higher than that in diabetic patients without DPN and that symptoms of both anxiety and depression commonly coexist in patients with DPN (Selvarajah et al., 2014a). Furthermore, patients with long-standing diabetes have been shown to be associated with impairments in acquisition and retrieval processes of spatial memory (Patel and Udayabanu, 2013). Therefore, one might speculate that decreased regional network parameters in the pars orbitalis and entorhinal cortex may be the neuropathological basis of the impairments in emotion regulation and spatial memory in patients with DPN.

### Network Hub Analysis

The DPN patients and HCs also differed in the number and distribution of network hubs. Four network hubs were found in HCs, whereas six hubs were identified in DPN patients. Some hubs in HCs are not retained in DPN patients, such as the left pars orbitalis and left rostral anterior cingulate cortex. In our study, the DPN patients showed significantly decreased cortical thickness in the pars orbitalis and cingulate cortex, suggesting that the disappearance of these hubs might arise from cortical thinning in these regions. In fact, the pars orbitalis and anterior cingulate cortex are functionally connected, and both participate in emotion regulation (Du et al., 2020). As such, alterations in these brain regions may underlie the emotion regulation impairments in DPN patients, which are consistent with the alterations in regional network parameters.

Furthermore, we found some new hubs in DPN patients, mainly in the prefrontal gyrus, banks of the superior temporal sulcus, insula cortex and fusiform gyrus, indicating a more central role of these regions in patient with DPN. On the one hand, the appearance of these hubs may occur as a result of pain and cognitive impairment. Functionally, the insula cortex is implicated in the sensory-discriminative and affective-motivational aspects of pain processing, whereas the frontal cortex is related to affective-motivational and anticipational components of pain (Burgmer et al., 2009; Watson, 2016). The insular cortex and prefrontal cortex are linked and activated during the processing of painful stimuli and are thus considered key regions of a network named the pain matrix (Ohara et al., 2008; Davis and Moayedi, 2013). The banks of the superior temporal sulcus are involved in various cognitive functions, such as audiovisual integration, as well as motion, speech, and face processing, whereas the fusiform gyrus, located on the ventral occipitotemporal surface, is selectively engaged in face recognition (Ghuman et al., 2014; Yue et al., 2020). Hence, the appearance of new hubs in these brain areas indicated that the alterations in the central nervous system of patients with DPN may involve changes in both pain-related and cognition-associated cerebral regions. This finding is consistent with previous task-fMRI studies, which showed stronger activation in these brain areas in the patients of DPN during thermal stimulation (Li et al., 2018). On the other hand, the appearance of these hubs may represent compensatory recruitment for decreased cortical thickness of these or other brain regions to maintain proper brain functioning, given that our study has shown significant cortical thinning in the prefrontal cortex, insular cortex, fusiform gyrus, and temporal lobes of DPN patients. However, the exact neural mechanisms underlying the finding of these new hubs in DPN patients remain unknown and need further exploration in the future.

### Limitations

There are some limitations for our research. Firstly, this is a cross-sectional study and did not consider the network topology alterations over time. Future longitudinal studies are warranted to unravel the dynamic pattern of abnormal brain networks in the DPN patients. Secondly, the medication of DPN patients was not completely consistent so medication confounding effects may exist. Therefore, the effect of medication should to be investigated in future studies. Thirdly, we did not investigate the correlation between network measurements and individualized clinical or neuropsychological variables, because the structural covariance networks are constructed at the group level. Future individualized network analyses are warranted to further pursue this issue.

## Conclusion

In the present study, we examined the topological alterations in the structural covariance networks in DPN patients compared with HCs. The structural covariance networks of patients with DPN showed a significantly increased characteristic path length, clustering coefficient, transitivity, and modularity, suggestive of inefficient global integration and increased local segregation. These results contribute novel insights into the pathophysiological mechanisms underlying alterations in the central nervous system of patients with DPN.

## Data Availability Statement

The original contributions presented in the study are included in the article/Supplementary Material, further inquiries can be directed to the corresponding author/s.

## Ethics Statement

The studies involving human participants were reviewed and approved by the Medical Research Ethics Committee of Xiangya Hospital, Central South University. The patients/participants provided their written informed consent to participate in this study.

## Author Contributions

FY and MQ analyzed and interpreted data and wrote and reviewed the manuscript. WL and JW reviewed the manuscript. YZ, LZ, WX, and JT prepared the data and reviewed the manuscript. WL and YZ designed the research, analyzed and interpreted the data, and reviewed the manuscript. All authors read and approved the final manuscript.

## Conflict of Interest

The authors declare that the research was conducted in the absence of any commercial or financial relationships that could be construed as a potential conflict of interest.

## Footnotes

1 http://surfer.nmr.mgh.harvard.edu/fswiki/CorticalParcellation

2 http://www.math.mcgill.ca/keith/surfstat/
