# Supplementary material for: Aberrant Brain Network Integration and Segregation in Diabetic Peripheral Neuropathy Revealed by Structural Connectomics
Source: Front Neurosci. 2020 Dec 4;14:585588. doi: 10.3389/fnins.2020.585588 (PMC7746555; doi:10.3389/fnins.2020.585588)
Supplement: Supplementary file 1 [file Data_Sheet_1.PDF]

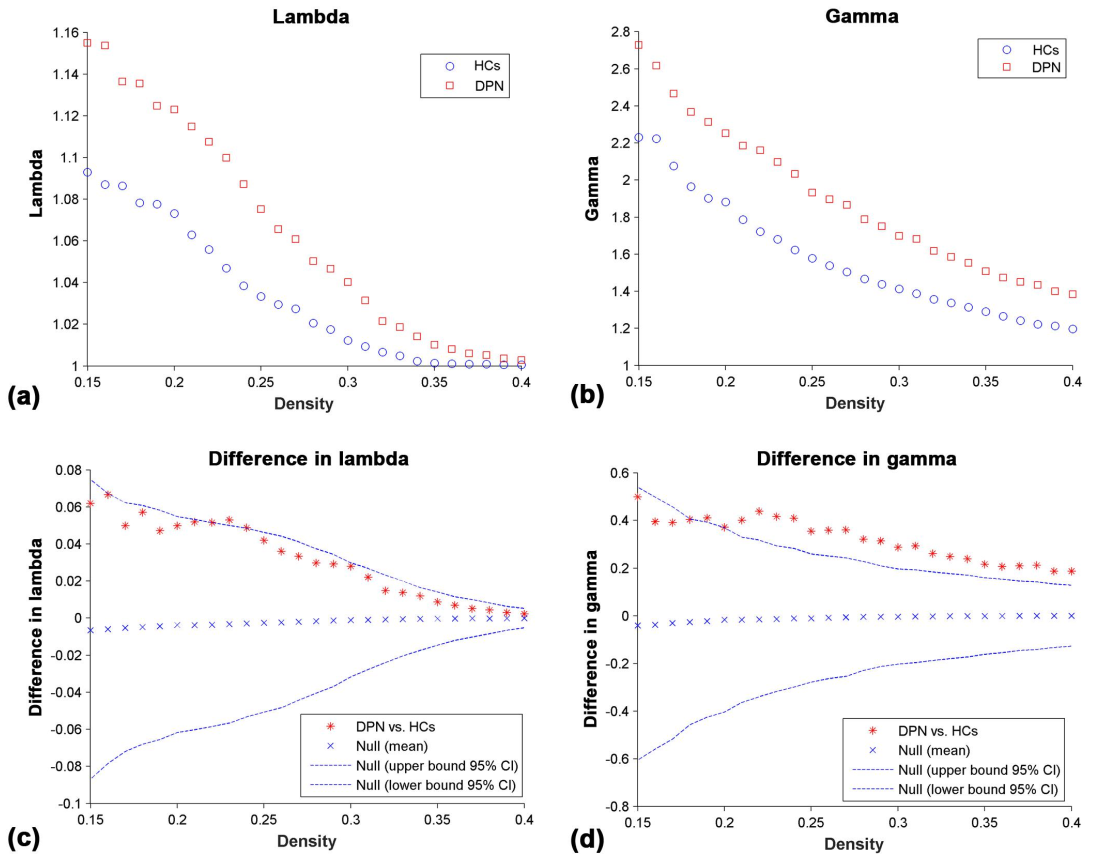

Figure S1 Changes in lambda (a) and gamma (b) as a function of network density. Between-group differences in lambda (c) and gamma (d) as a function of network density.

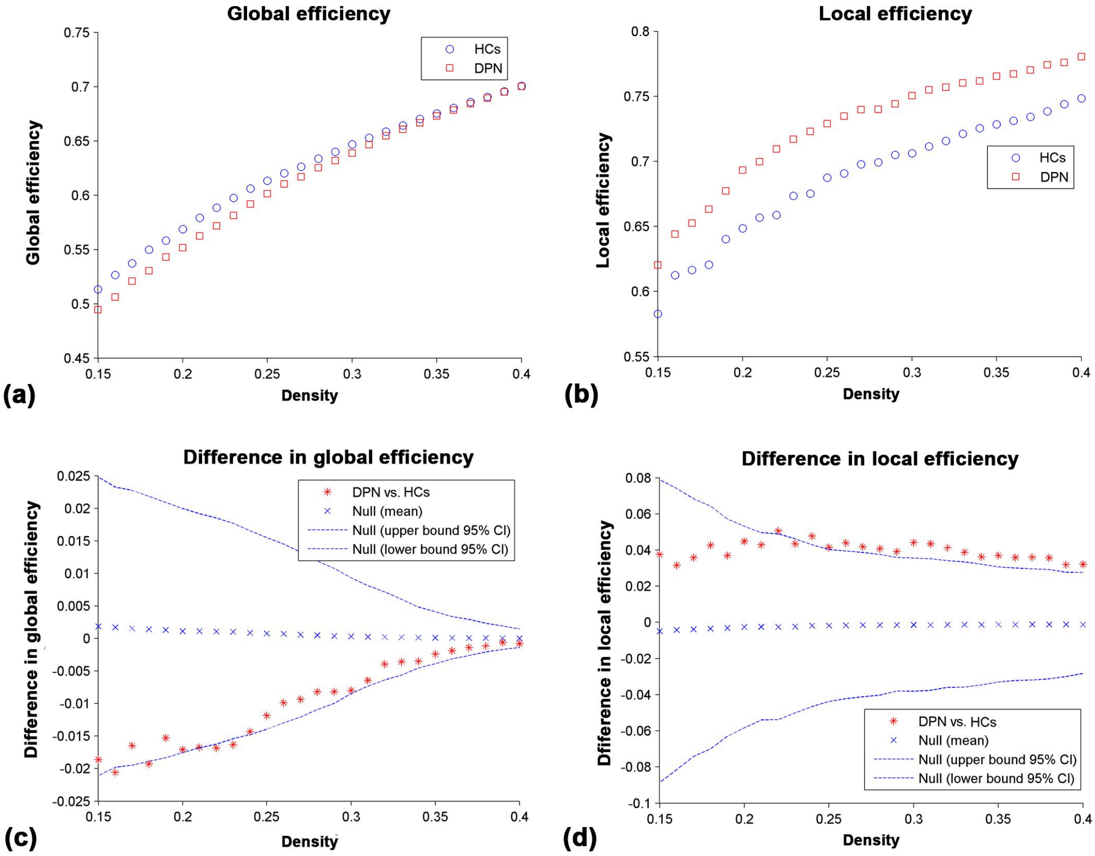

Figure S2 Changes in global efficiency (a) and local efficiency (b) as a function of network density. Between-group differences in global efficiency (c) and local efficiency (d) as a function of network density.
